# The influence of air pollution on residents’ outdoor exercise participation behaviour: Evidence from China Family Panel Studies

**DOI:** 10.1371/journal.pone.0270994

**Published:** 2022-08-30

**Authors:** Enkai Guo, Huamei Zhong, Jing Li, Yang Gao, Jie Li, Zhaohong Wang

**Affiliations:** 1 College of P.E and Sports, Beijing Normal University, Beijing, China; 2 School of Physical Education and Sport Science, Fujian Normal University, Fuzhou, China; Chung Shan Medical University, TAIWAN

## Abstract

Using data from China Family Panel Studies (CFPS) and based on the Probit and Tobit models, this study investigates the impact of air pollution on residents’ outdoor exercise behaviour from the microscopic level. Specifically, this study examined the effects of PM2.5 index changes on residents’ decision to participate in outdoor exercise and the duration of outdoor exercise participation. The empirical results show that the increase of PM2.5 index has a significant inhibitory effect on residents’ participation in outdoor exercise, and has passed the robustness test and endogeneity test. Further testing found that the inhibitory effect was significantly different between urban and rural areas, and in the central, north-eastern and western regions where economic development was relatively backward, the conclusion that air pollution inhibited residents’ outdoor exercise behaviour still holds true. However, the level of air pollution had no significant effect on the outdoor exercise behaviour of residents in the eastern region. So, while air pollution discourages residents from participating in outdoor exercise, the results are more applicable to less economically developed areas.

## I. Introduction

In recent years, with the intensification of environmental problems such as air pollution, residents’ enthusiasm for exercise participation and physical health have been greatly challenged. Studies have pointed out that particulate matter in air pollutants, including PM2.5 and PM10, has caused more than 4.2 million premature deaths worldwide [1]. In addition, due to the increased ventilation during exercise, the lung deposition of air pollutants increases, which increases the incidence of respiratory and cardiovascular system diseases, such as asthma [2–4]. However, there are also studies showing that long-term regular exercise can protect against the harmful effects of air pollution. As one study found, there is a correlation between habitual exercise and a reduction in premature deaths from air pollution [5]. In the experiment of pneumonia indicators in mice, it was also found that regular exercise is beneficial to the reduction of pneumonia indicators [6]. Given that both physical activity and air pollution are major factors affecting health, the key question of this study arises: “If air pollution is severe, will we give up regular outdoor physical activity?” In fact, in countries with more severe air pollution such as China, residents usually face this problem.

Since the 20th century, China’s economy has developed rapidly, and economic growth at the expense of the environment has eventually led to aggravation of environmental problems, among which the air quality has deteriorated significantly due to the increase in particulate matter emissions [7]. However, under the circumstance that the quality of the atmospheric environment is not effectively controlled, exercising in polluted air may not protect the health of residents, resulting in a decrease in residents’ participation in outdoor exercise. In China, the rise of outdoor exercise and the promotion of national fitness activities have increased the demand for residents to participate in exercise [8]. Based on that situation, China has issued the "Healthy China 2030" Plan Outline, which aims to strengthen residents’ exercise participation and promote physical health in terms of ecological environment governance and lifestyle changes. Under the guidance of cultural and policy norms, outdoor exercise are still sought after by Chinese residents, and places such as parks, squares, and streets are usually filled with residents who exercise daily [9]. Therefore, on the basis of forming a fixed exercise habit, it is difficult for a large number of residents who often participate in outdoor exercise to switch to indoor exercise because of air pollution [10]. That is to say, for people who exercise regularly, it is inevitable to exercise in an environment where indicators such as PM2.5 exceed the standard.

At present, there is not much research on how air pollution affects outdoor exercise behaviour, and the existing research has certain shortcomings. At present, most of the researches affecting outdoor exercise behaviour are cut from the perspective of exercise facilities, mainly including the quality of exercise facilities and the environment where the exercise facilities are located. On the one hand, high-quality exercise facilities are unique and stringent in terms of energy consumption, materials used, and comfort requirements [11]. China’s "Basic Configuration Standards for National Public Exercise Facilities" formulated construction standards for various exercise facilities. For example, there should be no less than 12 exercise facilities in a large-scale national fitness activity centre, with a construction area of 8,000–12,000 square metres and an indoor and outdoor exercise area of no less than 5,000 square metres. In addition, construction materials need to be environmentally friendly, such as wooden floors, synthetic materials, and artificial natural turf, to ensure the quality of the mass exercise environment. The number of exercise facilities is also proportional to the residents’ outdoor exercise participation behaviour. Surveys show that residents living in counties with more exercise facilities are more likely to engage in physical activity and have higher levels of well-being [12]. Additionally, quality monitoring of existing exercise facilities is also an important link. For example, swimming facilities require regular water quality management, including humidity, disinfection, and facility maintenance [13].

On the other hand, air quality in the location of exercise facilities is also an important factor affecting human health and exercise performance [14]. During moderate-to-high-intensity exercise, people mostly choose to breathe air through their mouths, bypassing the mechanism of filtering soluble particles in the nasal cavity [15]. Therefore, during exercise, with the increase in air flow, more pollutants will enter the respiratory tract, which is not conducive to human health. If the air quality in the exercise environment is poor, people may reduce the frequency of exercise, and countries or regions with relatively good air quality will also have relatively high exercise participation. This corresponds to the fact that many Nordic countries have a high level of exercise participation; for example, according to a survey from the European Commission’s Directorate General for Education and Culture (DGEAC), the exercise participation rate of residents of Sweden, Denmark and Finland reached more than 60% in 2014 [13].

So far, most relevant studies have been conducted in Western countries [16], such as the use of data released by the US Centres for Disease Control and Prevention to examine the relationship between air pollution and physical activity [17,18]. Most studies have reached a similar conclusion that air pollution is significantly associated with a reduction in physical activity. Air quality in Western countries is generally better than in China, however, there are relatively few studies on air quality and outdoor exercise behaviour in China, mainly due to the difficulty of obtaining national data. Most of the existing studies are based on individual cases or a few residents, which lack a certain degree of representativeness. For example, some studies use software apps to obtain exercise data of 389 users and conduct empirical tests with small samples [10]. In addition, some studies have shown an inverse relationship between environmental pollution and outdoor physical activity in adults [19], and that environmental pollution is associated with increased sedentary behaviour and increased sleep time [20]. Thus, there has not yet been a study that comprehensively assessed the impact of ambient air pollution in China on the full-range exercise behaviour of different geographic regions or different age groups through long-term follow-up surveys. Consequently, this study proposes two research hypotheses.

**H1:** The higher the level of air pollution, the less likely residents are to choose to participate in exercise.
**H2:** The higher the air pollution, the less time residents spend participating in exercise.

That is to say, this study makes up for the insufficiency of related research through a follow-up survey across years and covering all of China, and uses the Probit and Tobit models to solve the research question: Does air pollution affect outdoor exercise decision-making? How does air pollution affect outdoor exercise behaviour?

## II. The method and dataset

### 1. The statistical models

Available studies have shown that there is a linear relationship between air pollution and residents’ outdoor exercise participation behaviour. Among them, residents’ outdoor sports participation behaviour may be related to residents’ personal characteristics, such as gender, age, education level, employment status, family status, etc. In addition, risk factors for certain diseases, such as obesity [21] and smoking [22], can also alter outdoor exercise participation in potential or pre-existing populations. However, certain factors, such as rainfall and pollutant emissions, can affect air pollution levels and, in turn, residents’ outdoor exercise behaviours. Therefore, in this study, combining Hypothesis 1 and Hypothesis 2, all the above-mentioned variables were included as control variables, and the following research model was constructed:

$${Exercise2}_{ipt}=\beta_{0}+\beta_{1}{PM25}_{pt}+\sum \beta_{2}X_{ipt}+\sum \beta_{3}\mu_{pt}+V_{p}+T_{t}+\varepsilon_{ipt}$$ (1)

$${Exercise\;last}_{ipt}=\beta_{0}+\beta_{1}{PM25}_{pt}+\sum \beta_{2}X_{ipt}+\sum \beta_{3}\mu_{pt}+V_{p}+T_{t}+\varepsilon_{ipt}$$ (2)

Model 1 is a binary choice model, where Exercise2 indicates whether to choose to participate in exercise. The dependent variable is a binary variable, so the Probit model with higher generality can better describe the results of this study. Model 2 is the Tobit estimation model, where Exercise last is the length of time residents participate in exercise. Since in the adopted data, there are many individuals whose outdoor exercise time is 0, the Tobit model is more suitable. Both models explore the impact of urban environmental pollution on individual outdoor exercise behaviours from a microscopic perspective. In these two models, subscripts p and t denote the province and year of the sample at the time of the survey, respectively, while the subscript i represents the sample individual. The independent variable: PM25 denotes the PM2.5 index. In the subsequent robustness test, the AQI index will be used to replace PM2.5 to test the robustness of the model. X_ipt_ is a series of control variables at the individual level, including gender, age, marital status, education level, employment status, BMI, etc. μ_pt_ is a series of control variables at the provincial level, such as precipitation and nitrogen oxide emissions. V_p_ is the province fixed effect, T_t_ is the year fixed effect, and ε_ipt_ is the random interference term. All regressions will be tested by STATA.

### 2. Ethics statement

CFPS projects are research projects involving people. In order to ensure that the rights and interests of the respondents participating in the project are protected to the greatest extent, an application for ethics review or continuous review is regularly submitted to the "Peking University Biomedical Ethics Committee", and the corresponding data collection work is carried out when the ethics review is approved. Since the CFPS project is a continuous follow-up investigation project, after the initial review is passed, we submit an application for continuous review to the Ethics Review Committee in subsequent years. The ethics review batch number of the CFPS project will not change due to different investigation rounds, and the review batch number is unified as: IRB00001052-14010.

In addition, before the start of the investigation, the researchers will first obtain the informed consent form of the subjects through paper means. In addition, when interviewing information about minors, it is conveyed by parents or guardians, and their consent has been obtained. Therefore, at the beginning of this study, all data were completely anonymized.

### 3. The variables and dataset

The data for this study were obtained from the China Family Panel Studies (CFPS), which focuses on the development and changes of China’s society, economy, population, health and education by collecting data from three dimensions: individual, family and community. The CFPS adopts various forms of questionnaires, such as long, short, pick-up, and telephone interviews, to provide a data basis for relevant academic research and public policy formulation. CFPS is hosted by Peking University China Social Science Survey Centre (ISSS), assisted by universities and institutions such as the University of Michigan Social Research Centre, and has officially been visited since 2010, covering 95% of all provinces in China [23,24]. The CFPS covers a large proportion of China’s population, and the obtained data are random and representative, which can better reflect the real situation of China’s population. Therefore, this study uses the data from this database in 2014, 2016 and 2018 to better demonstrate the degree of influence of air quality on residents’ outdoor exercise participation behaviour from a microscopic level.

The descriptive statistics and calculation basis of the variables are shown in Table 1. According to the existing research basis, the main dependent variable of this study is Exercise last, which represents the duration of residents participating in outdoor exercise. Based on this variable, a binary variable, Exercise2, is calculated, that is, whether residents participate in outdoor exercise. To evaluate the accuracy of the research model, this study selected outdoor exercise frequency and the frequency of outdoor exercise participation as surrogate variables in the robustness test. The independent variable of this study is the PM2.5 index, and the data are from China’s air quality online monitoring and analysis platform. There are three aspects to the controlled variable. The first is sociodemographic aspect, including gender, age, marital status, education level, employment status, urban or rural, and family size. The second is the economic aspect, mainly the average monthly net income. The third is subjective perception, mainly the perception of self-health status, which includes five levels, ranging from very healthy to very unhealthy.

**Table 1:** Descriptions of variables.

Variable	Description	Coding values
Dependent variable		
Exercise fre	Frequency of outdoor exercise participation	Divide into 1–7 categories based on exercise frequency
Exercise1 (E1)	Whether or not there is participation in outdoor exercise	Yes = 1, no = 0 (calculated by exercise fre)
Exercise last	Length of time of participation in outdoor exercise a week	Measured in hours
Exercise2 (E2)	Whether or not there is participation in outdoor exercise	Yes = 1, no = 0 (calculated by exercise last)
Independent variable		
PM25	The average PM2.5 index of the month where the interviewer was interviewed	
AQI	The average Air Quality Index of the month where the interviewer was interviewed	
Controlled variables		
Gender	Gender	Male = 1, female = 0
Age	Age	Measured in years
Married	Marital status	Married = 1, unmarried = 0
Education	Years of education	Measured in years
Employ	Employment status	Employed = 1, other = 0
Urban	The residence of the interviewee	Urban = 1, rural = 0
Family count	The number of family members living together	
Monthly income	The logarithm of monthly net income	Measured in Chinese yuan
Debts	Mortgage and various financial loans	Debts = 1, no debts = 0
Health care expenditure	Expenses for medical treatment, purchase of sporting goods, health care products, etc.	Measured in Chinese yuan
Health condition	The interviewer’s personal perception of his or her health	Measured on a scale of 1 (very healthy) to 5 (very unhealthy)
Smoke	Have you smoked in the past month	Yes = 1, no = 0
BMI	Body mass in dex (Height/Weight^2)	Measured on a scale of 1 (underweight, obese), 2 (overweight), 3 (normal)
Precipitation	Total precipitation by province (mm)	
Nitrogen oxides	Total nitrogen oxide emissions (10,000 tons)	

### 4. Descriptive statistics

Tables 2 and 3 describe the descriptive statistical results of the selected variables in this study in 2014, 2016 and 2018. The results show that from 2014 to 2018, the frequency of per capita monthly participation in exercise increased from 1.66 to 5.92, and the possibility of residents choosing to participate in exercise also increased from 37.25% to 49.34%. It can be seen that with the advancement of the "National Fitness" strategy, China’s exercise population has increased significantly, and the frequency of exercise participation is higher. With the passage of time, the overall air pollution level in China has gradually decreased (the PM2.5 index dropped from 46.54 to 19.62, and the AQI index dropped from 75.01 to 63.00), which means that the effect of environmental governance has become increasingly prominent, and the air quality has improved. Additionally, the average monthly income of residents is also on the rise, providing good economic support for residents to participate in outdoor exercise. The increase in health care expenditure year by year reflects the improvement of residents’ awareness of health care and provides protection for participation in outdoor exercise activities through the purchase of exercise equipment. The increase in the proportion of people who consider their health status to be above good (questionnaire score of 3 and below), combined with the increase in the frequency of residents participating in outdoor exercise and the increase in average monthly income, provides a factual basis for the increase in the health level and life expectancy of Chinese residents.

**Table 2:** Descriptive statistics of continuous variables.

Variable	2014 (n = 7933)		2016 (n = 4339)		2018 (n = 9104)		Total (n = 21376)	
	M	SD	M	SD	M	SD	M	SD
Exercise fre	1.662	2.633	1.859	2.715	2.307	3.080	1.977	2.862
Exercise last	2.393	6.080	2.955	7.624	3.486	7.917	2.973	7.242
PM25	46.536	19.705	39.517	18.905	29.373	14.774	37.802	19.203
AQI	75.009	23.831	75.170	21.305	71.374	19.503	73.494	21.638
Age	39.015	12.391	35.671	12.649	40.563	12.553	38.995	12.643
Education	9.688	4.142	10.190	4.172	10.129	4.258	9.978	4.204
Family count	4.228	1.808	4.091	2.005	3.978	2.113	4.094	1.986
Monthly income	7.420	0.982	7.471	1.117	7.731	0.977	7.563	1.019
Health care expenditure	7.590	1.415	7.694	1.434	7.820	1.360	7.709	1.400
Precipitation	923.702	492.166	1146.33	664.355	976.473	467.343	991.37	528.34
Nitrogen oxides	86.575	42.311	64.076	34.729	54.575	29.640	68.38	38.610

**Table 3:** Descriptive statistics of categorical variables.

Variable	2014 (n = 7933)		2016 (n = 4339)		2018 (n = 9104)		Total (n = 21376)	
	Frequency	%	Frequency	%	Frequency	%	Frequency	%
Not exercising (from E1)	4976	62.75	2517	58.01	4711	51.75	12207	57.10
Exercising (from E1)	2954	37.25	1822	41.99	4393	48.25	9169	42.90
Not exercising (from E2)	4976	62.76	2517	58.01	4723	51.88	12219	57.16
Exercising (from E2)	2954	37.24	1822	41.99	4381	48.12	9157	42.84
Female	3128	39.43	1938	44.66	3675	40.37	8741	40.89
Male	4805	60.57	2401	55.34	5429	59.63	12635	59.11
Unmarried	1251	15.77	1075	24.78	1390	15.27	3716	17.38
Married	6682	84.23	3264	75.22	7714	84.73	17660	82.62
Unemployed	529	6.67	324	7.47	600	6.59	1453	6.80
Employed	7404	93.33	4015	92.53	8504	93.41	19923	93.20
Rural	3224	40.64	1784	41.12	3655	40.15	8663	40.53
Urban	4709	59.36	2555	58.88	5449	59.85	12713	59.47
No debts	6729	84.82	3519	81.10	5558	61.05	15806	73.94
Debts	1204	15.18	820	18.90	3546	38.95	5570	26.06
Health condition								
Excellent	1322	16.66	779	17.95	1342	14.74	3443	16.11
Very good	1846	23.27	900	20.74	1553	17.06	4299	20.11
Good	3154	39.76	1785	41.14	4325	47.51	9264	43.34
Fair	1038	13.08	581	13.39	1116	12.26	2735	12.79
Poor	573	7.22	294	6.78	768	8.44	1635	7.65
No smoking	5013	63.19	2937	67.69	5836	64.10	13787	64.49
Smoking	2920	36.81	1402	32.31	3268	35.90	7589	35.51
BMI								
Underweight and obese	1153	14.53	3280	75.59			5783	27.05
Overweight	2123	26.80	218	5.02			5036	23.56
Normal	4654	58.67	841	19.38			10557	49.39

## III. Results

### 1. Benchmark regression results

The research was carried out using the data in CFPS, and the benchmark regression results of Formulas (1) and (2) are shown in Table 4. Before performing the benchmark regression, this study also conducted a variance inflation factor (VIF) test. The results showed that the VIF values of all variables in each year were less than 3, which was much lower than 10, successfully avoiding the multicollinearity of the variables [25]. Research shows that the PM2.5 index have a significant negative impact on residents’ outdoor exercise participation decision-making and participation duration, and the coefficients of the independent variables are all significant at the 1% level. Both the Probit model and the Tobit model confirmed this result.

**Table 4:** Benchmark regression results.

Variable	Exercise2		Exercise last	
	(1) Probit	(2) Probit	(3) Tobit	(4) Tobit
pm25	-0.002*** (0.0006)	-0.003*** (0.0006)	-0.025*** (0.006)	-0.028*** (0.007)
Gender		0.134*** (0.023)		1.321*** (0.250)
Age		0.017*** (0.001)		0.198*** (0.011)
Married		-0.302*** (0.029)		-2.841*** (0.313)
Education		0.072*** (0.003)		0.564*** (0.032)
Employ		-0.077** (0.036)		-0.251 (0.359)
Urban		0.202*** (0.020)		1.255*** (0.234)
Family count		-0.019*** (0.005)		-0.067 (0.058)
Monthly income		0.002 (0.010)		-0.344** (0.135)
Debts		0.024 (0.021)		-0.033 (0.238)
Health care expenditure		0.015** (0.007)		0.202*** (0.078)
Health condition		-0.058*** (0.009)		-0.619*** (0.102)
Smoke		-0.159*** (0.007)		-0.755*** (0.263)
BMI		0.015 (0.012)		0.291** (0.136)
Precipitation		-0.00004 (0.00009)		-0.001*** (0.0002)
Nitrogen oxides		-0.0002 (0.001)		-0.007** (0.003)
_cons	-0.097*** (0.088)	-1.202*** (0.152)	-4.363*** (0.368)	-12.554*** (1.353)
Year	Control	Control	Control	Control
Province	Control	Control	Control	Control
Adj/Pseudo R^2^	0.017	0.070	0.002	0.012
N	21376	21376	21376	21376

Note

(1) *p < .1. **p < .05. ***p < .01.

(2) Robust standard error for t-statistics in parentheses.

The results of the Probit model showed that when other variables are controlled, an increase in the PM2.5 index by one unit will reduce the possibility of residents choosing to participate in outdoor exercise by 0.2%. Therefore, Hypothesis 1 is proven to hold. The results of the Tobit model regression showed that when other variables were controlled, the duration of residents participating in outdoor exercise decreased by 0.023 hours for each unit increase in the PM2.5 index. Therefore, Hypothesis 2 is proven to be true.

In addition, the regression results of the controlled variables in the two models are also roughly the same as those of the existing studies. First, at the sociodemographic level, age and education level are positively proportional to residents’ decision to participate in outdoor exercise and the duration of outdoor exercise, which is consistent with the relevant research results in France, Germany and Italy [26]. Male, unmarried, unemployed, urban residents and non-smoker are also relatively more motivated to participate in exercise. However, in terms of family structure, the larger the family population, the less likely residents are to participate in outdoor exercise [12,27,28]. Second, at the economic level, although a higher income and health care expenditure are related to a greater likelihood of participating in outdoor exercise [29], the middle-income groups spend significantly less time playing outdoor exercise than low-income groups. The findings are similar to the results of a survey in England, where middle-income groups spend less time participating in outdoor exercise because of limited discretionary time [30]. In terms of financial debts, individuals with debts are more likely to participate in outdoor exercise, form debt accumulation in the form of gambling, betting, etc. [31]. Debt-free residents, on the other hand, have longer exercising times because they are free to spend their time and enjoy the charm of outdoor exercise without the pressure of external debts. Finally, at the self-perceived level, the better the self-perceived health level, the stronger is the willingness to participate in outdoor exercise and the longer is the outdoor exercise participation time [32].

### 2. Robustness check

The dependent variable used in the benchmark regression of this study is the length of residents’ outdoor exercise participation time, but only measuring residents’ outdoor exercise participation behaviour with this variable will inevitably lead to a reduction in the reliability of the model. Therefore, to verify the rationality of the model, this study introduced another dependent variable with a similar meaning, namely, the frequency of residents’ participation in outdoor exercise, to replace the original dependent variable. Likewise, in the robustness test, the binary variables in Model 1 are also scaled according to the newly introduced dependent variable. Since the frequency of outdoor exercise is an ordered face change, column (2) is analysed and verified using the Ologit model. In addition, as mentioned above, this study also introduced the AQI indicator to replace the PM2.5 index to ensure the robustness of the research model.

The robustness test results are shown in Table 5. Columns (1) and (2) in Table 5 show that the PM2.5 index have a negative impact on the frequency of residents’ participation in outdoor exercise, and they are still significant at the 1% significance level. Column (3) and (4) show that after other variables are controlled, the AQI index also have a negative impact on the length of time of participation in outdoor exercise a week. In view of this, the results of the robustness test showed that in the context of replacing the dependent and independent variables, the research model used in this study is still robust, and the research results have certain reference significance.

**Table 5:** Robustness check results.

Variable	Exercise1	Exercise fre	Exercise2	Exercise last
	(1) Probit	(2) Ologit	(3) Probit	(4) Tobit
pm25	-0.003*** (0.0006)	-0.004*** (0.001)		
AQI			-0.003*** (0.0006)	-0.024*** (0.007)
Controlled variables	Control	Control	Control	Control
Year	Control	Control	Control	Control
Province	Control	Control	Control	Control
Adj/Pseudo R^2^	0.070	0.033	0.070	0.013
N	21373	21376	21376	21376

Note

(1) *p < .1. **p < .05. ***p < .01.

(2) Robust standard error for t-statistics in parentheses.

### 3. Endogenous test

Although a series of control variables and a year fixed effect were included in the regression model, endogeneity problems and estimation biases caused by omitted variables were minimized. But there may still be third-party factors that can affect core explanatory variables, such as the PM2.5 index, and bias the estimated coefficients. Therefore, this study attempts to overcome by instrumental variable method.

Under the analysis framework of this paper, the qualified instrumental variables need to meet the exogenous conditions and be highly correlated with the air pollution index, but not with the outdoor exercise behaviour of residents. The river area (thousand hectares) of each region can undoubtedly meet the above criteria. As an important indicator to measure the level of urban greening, river area will naturally affect the degree of air pollution in the city. In addition, river area was not directly related to residents’ outdoor exercise behaviour. Therefore, river area can be used as an instrumental variable for air pollution. This study summarizes the area of rivers in various regions through the "China Environmental Statistical Yearbook" over the years.

Table 6 presents the estimated results of the instrumental variables. The regression results of the first stage show that the river area is significantly negatively correlated with air pollution, indicating that the expansion of the river area is conducive to promoting the improvement of air quality. And the p-value of the first-stage regression F test is close to 0, rejecting the null hypothesis, indicating that there is no problem of weak instrumental variables. The second-stage regression results are reported in columns (2) and (4). The estimated results are -0.159 and -0.098, respectively, and both are significant at the 1% confidence level. These estimates suggest that air pollution in China significantly affects residents’ outdoor exercise behaviour, and the results are robust.

**Table 6:** Instrumental variable regression results.

Variable	pm25	Exercise Last	AQI	Exercise Last
	(1) first stage	(2) IVTobit	(3) first stage	(4) IVTobit
pm25		-0.159*** (0.048)		
AQI				-0.098*** (0.029)
River area	-0.015*** (0.0007)		-0.023*** (0.0008)	
Controlled variables	Control	Control	Control	Control
Year	Control	Control	Control	Control
N	21376	21376	21376	21376
Adj/Pseudo R^2^	0.310		0.034	
F	535.03		598.14	

Note

*p < .1. **p < .05. ***p < .01.

### 4. Heterogeneity test

Given the regional differences in air pollution [26,30], this study examines the heterogeneity of the effects of air pollution on outdoor exercise behaviour from the perspective of different regions. The samples are divided into four groups according to the economic belts officially announced by the Chinese government, namely, the eastern region, the central region, the north-eastern region and the western region. The eastern region includes 10 provinces (cities), including Beijing, Tianjin, Hebei, Shanghai, Jiangsu, Zhejiang, Fujian, Shandong, Guangdong and Hainan. The central region includes Shanxi, Anhui, Jiangxi, Henan, Hubei and Hunan Provinces. The western region includes 12 provinces (autonomous regions and municipalities), encompassing Inner Mongolia, Guangxi, Chongqing, Sichuan, Guizhou, Yunnan, Tibet, Shaanxi, Gansu, Qinghai, Ningxia and Xinjiang. The northeast region includes Liaoning, Jilin and Heilongjiang. The test results are shown in Table 7.

**Table 7:** Heterogeneity test results.

Variable	Exercise Last					
	(1) Eastern	(2) Central	(3) North-eastern	(4) Western	(5) Urban	(6) Rural
pm25	0.004 (0.013)	-0.024* (0.012)	-0.058** (0.023)	-0.056*** (0.020)	-0.030*** (0.009)	-0.020** (0.015)
_cons	-12.552*** (2.758)	-18.643*** (3.646)	-31.074*** (10.160)	-14.493*** (8.149)	-9.681*** (1.673)	-17.072*** (4.096)
Controlled variables	Control	Control	Control	Control	Control	Control
Year	Control	Control	Control	Control	Control	Control
Province	Control	Control	Control	Control	Control	Control
Adj/Pseudo R^2^	0.012	0.018	0.015	0.013	0.013	0.012
N	8305	5272	2761	5038	12713	8663

Note

(1) *p < .1. **p < .05. ***p < .01.

(2) Robust standard error for t-statistics in parentheses.

The test results showed that in areas with lower economic development levels, air pollution has a more significant inhibitory effect on residents’ exercise participation, which is clearly in line with theoretical expectations. In the central, north-eastern and western regions, where economic development is relatively backwards, for every unit of increase in the PM2.5 index, the length of residents’ participation in exercise decreases significantly by 0.024, 0.058 and 0.056 hours, respectively. However, the data show that in the more economically developed eastern regions, air pollution has no significant effect on residents’ outdoor exercise behaviour.

In addition, this study also examines the heterogeneity of its influence effect from the perspective of urban-rural differences. The result is in column (5) and (6). The results show that although the coefficient of air pollution is negative, there is still a certain significant difference in the degree of response between urban and rural areas. That is to say, in rural areas, residents’ outdoor exercise participation behaviour is more susceptible to air pollution.

## IV. Discussion and conclusion

### 1. Discussion

This study aims to explore the relationship between air quality and outdoor exercise behaviour, and provide a reference for residents to exercise outdoors under the condition of air pollution. To achieve this, we investigated the relationship between air quality and residents’ participation in outdoor exercise decisions and duration based on CFPS data. The results of the study showed that air quality was significantly associated with residents’ participation in outdoor exercise decision-making and duration. As hypothesized, as air pollution levels increased, residents were less likely to participate in outdoor exercise, and the duration of outdoor exercise gradually decreased. In terms of urban-rural differences, urban residents’ outdoor exercise behaviour is significantly more affected by air pollution than rural residents. In addition, from the perspective of economic regions, this study also found that only the central, north-eastern and western regions have significant differences, and the eastern region is not significant. Based on these results, it appears that air quality may influence residents’ outdoor exercise decisions and duration, but the effects of air pollution may be greatly diminished in more economically developed regions.

Although many studies have shown that exercising in a polluted environment can have many adverse health effects [3,33]. However, for residents living in more economically developed areas, giving up the habit of regular outdoor exercise, and its health benefits, is also a big challenge [34]. That is to say, how to balance the impact of exercise and air pollution on health, the key is to find out the critical value at which physical activity can reduce and increase the adverse effects of air pollution on physical health [35].

On World Health Day on April 7, 2019, the United Nations Environment Programme issued a statement and pointed out that air pollution has become the biggest threat to human health, causing an average of 6.5 million deaths each year. Similar to air pollution, participating in outdoor exercise can also affect physical health and pose a risk of death [33]. As a populous country, China has always had serious environmental problems. Therefore, research on the relationship between air pollution and outdoor exercise in China is important. According to the research data, in recent years, the overall air quality of China’s provinces has shown a downward trend, while the national exercise population has shown an increasing trend. The improvement of air quality has created conditions for the development of China’s outdoor exercise industry, and mountain outdoor exercise, such as marathons and bicycles, have gradually emerged [9]. Although the current air pollution problem is gradually improving, it may still take a long time to maintain the air quality at a level suitable for outdoor exercise. That said, residents who exercise outdoors in areas with relatively low air quality may still have higher health risks.

However, some studies had shown that in certain circumstances, the benefits of exercising in a polluted environment outweigh the disadvantages [36]. As Neidell (2004) found that, pollution is a potential mechanism by which socioeconomic status affects residents’ health [37]. Air pollution has a greater impact on the health behaviours of low-income people because they need to consider whether they can afford the health and economic costs of a polluted environment. For high-income people, just because of moderate air pollution, they are discouraged from outdoor exercises, and the sedentary state instead is more likely to increase the risk of chronic diseases such as obesity and diabetes. This means that residents living in more developed areas usually have higher economic incomes, and once they have started regular outdoor exercise, it is difficult to stop because of air quality problems. Therefore, it is necessary for the government or relevant organization to evaluate and publish the quality of local air pollution and its harm to the health degree. Then the government or relevant scholars can provide reasonable suggestions for residents, especially those living in areas with low economic development levels, on whether they can carry out outdoor sports, to maximize the health of the residents in the ensure.

### 2. Strengths and limitations

This study is the first to study the relationship between air quality and outdoor exercise behaviour in China based on a large amount of microscopic data. This study helps to complement the existing literature results, mainly including the following three advantages.

First, although international researchers have discovered the relationship between air quality and physical activity in Western countries such as the United States through large-scale data research [17]. However, at present, there are few relevant studies on China and other developing countries and areas with serious environmental pollution, mainly due to the difficulty in obtaining relevant data. By continuously tracking Chinese residents, CFPS has obtained a large amount of microscopic data, which is helpful for exploring the influence of air quality on outdoor exercise behaviour in areas with relatively severe environmental pollution. This has important practical significance for strengthening pollution control measures in severely polluted areas.

Second, previous studies have only explored the impact of air quality on outdoor exercise behaviour from the aspects of gender and activity patterns [10]. This is one-sided, and it is necessary to consider residents’ living conditions, personal income, and socio-demographic factors. For example, in economically developed areas, although the surrounding air quality is declining, they have relatively high per capita income, which is enough to support them to choose indoor fitness instead of outdoor exercise [38]. This is similar to the reason why the impact of air quality on outdoor exercise participation of urban residents is relatively lower than that of rural residents.

Third, most of the previous studies collected objective data, namely air quality indicators and physical activity indicators. This method usually ignores the interference of subjective health status and is prone to analytical errors. Because often, air pollution and outdoor exercise can have both beneficial and detrimental effects on the health of residents [39]. Therefore, when conducting research, it is necessary to control for health status [32,40]. This study selected residents’ subjective fitness level as a control variable, which adds to the reliability of our findings on the link between outdoor exercise and air quality.

However, this study still has some limitations. First, due to the time lag of the data, the data from 2014–2018 might not accurately reflect the current situation. However, due to the complexity of the data survey, the data for 2020 would only be released in early 2022, so real-time data were difficult to obtain. And the covid-19 epidemic around 2020 has had a certain inhibitory effect on the outdoor sports behaviour of Chinese residents, and even residents around the world. Therefore, future related research can incorporate the impact of the epidemic into the research model to enrich related research. In addition, from December 2020, the Chinese government clearly put forward the goal of peaking carbon emissions by 2030, and since then, environmental governance has been significantly increased. Therefore, the air pollution data starting in 2020 will have greater fluctuations than before, and future research should explore new analytical models to match the corresponding data. Second, the length of time spent outdoor exercise, the core explanatory variable in this study, was obtained by subjective measures and may be affected by memory errors. Therefore, future related research can be combined with applications that can record motion status to ensure the accuracy and objectivity of relevant data.

### 3. Conclusion

The increase in air pollution has reduced residents’ willingness to participate in outdoor exercise, because exercising in a polluted environment is not good for health. And this result still holds true for residents in urban, rural, and less developed areas. However, in economically developed areas, once residents have regular exercise habits, it is difficult to reduce the duration and frequency of outdoor exercise due to air pollution. In view of this study, there are still some limitations in the selection and acquisition of data. Therefore, after the release of the latest survey data, scholars can consider constructing a new research panel to deeply explore the relationship between air pollution and residents’ outdoor exercise participation behaviour.
